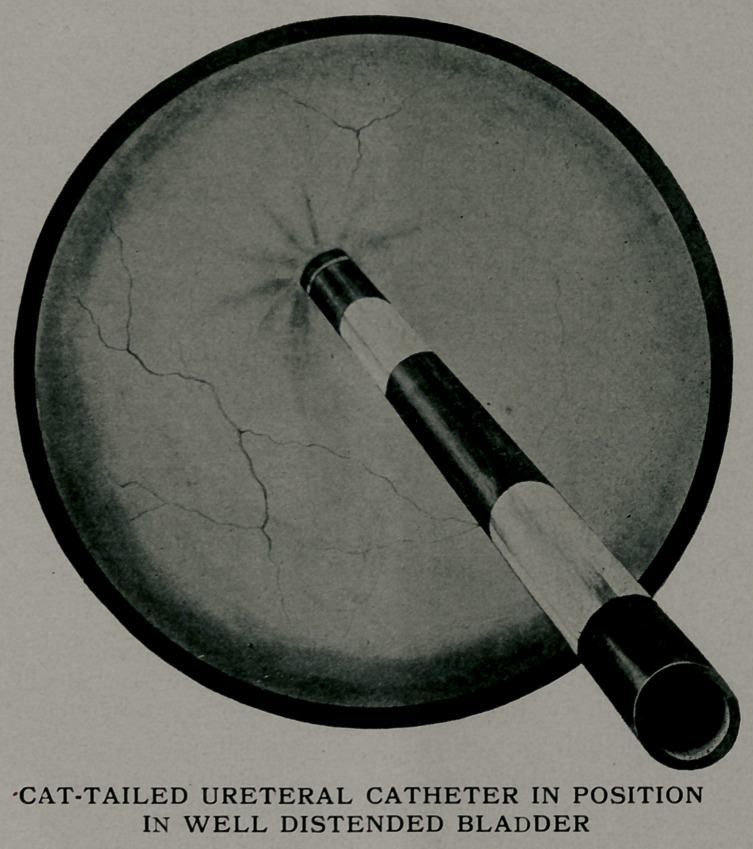# Simultaneous Catheterization of the Ureters

**Published:** 1910-12

**Authors:** Alfred L. Fowler

**Affiliations:** Atlanta, Ga.; Professor of Gento-Urinary Surgery in the Atlanta College of Physicians and Surgeons; Genito-Urinary Surgeon to the Grady (City) Hospital; Surgeon to St. Joseph’s Infirmary; Physician and Surgeon to United States Penitentiary Hospital; Member American Urological Association, Etc.; 928-029 Candler Building


### SIMULTANEOUS CATHETERIZATION OF THE URETERS.

BY ALFRED L. FOWLER, M. D., ATLANTA, GA.

*Professor of Gento-Urinary Surgery in the Atlanta College of Physicians and Surgeons; Genito-Urinary Surgeon to the Grady (City) Hospital; Surgeon to St. Joseph's Infirmary; Physician and Surgeon to United States Penitentiary Hospital; Member American Urological Association, Etc.*

The brilliant results in kidney surgery, now being reported from all over the world, are due largely to our greater precision in renal diagnosis. Ureteral catheterization has made this possible.

Before the advent of ureteral catheterism the principal reason for post-operative deaths were:

(1) Congenital absence of a second kidney, and

(2) A diseased kidney incapable of proper functioning capacity sufficient to preserve the patient after the operation.

Ureteral catheterism discloses if both kidneys are present, tells us from which kidney the pus comes, enables us to determine the functionating capacity of each kidney, points out from which kidney comes the hemorrhage, and with the wax-tipped catheter detects stone in the kidney pelvis and, lastly, stricture of the ureters.

BLACK URETERAL CATHETER, IN A MODERATELY DISTENDED BLADDER, IN LEFT URETER.

The bladder mucosa and ureteral openings, as viewed through the cystoscope, disclose a great amount of information to the trained eye. For this reason we study the bladder membrane well before engaging the catheters and passing them up to the kidney pelvis.

Catheterizing the ureters is exact, practical and safe, and out of more than a thousand cases that I have catheterized I have not observed a single instance where the slightest harm has come from the procedure.

In illustration of the practicability of the method, together with its usefulness, I cite you as follows:

A recent case, referred by Dr. A. P. Flowers, gave a history of a sudden attack coming on at night of painful and frequent micturition, without any assignable cause. The doctor treated him for cystitis, and as the condition failed to clear under appropriate treatment, he very rightly presumed his patient's kidneys were at fault. There was no pain in the kidney region. I cystoscoped him and found the mucosa on the right of the trigone injected, in well isolated areas, a beefy red with many patchy necrotic areas. The right ureter opening on this side dilated and contracted normally, as did its fellow, but was a little injected.

After passing the ureteral catheters to the kidney pelvis we found the separate specimens to show milk-tinted urine from right kidney and clear amber colored urine from left. "T B" bacilli were detected with miscroscope and the injected urine into abdominal cavity of guinea pigs killed them in four and five weeks and whose peritoneal cavities were found to be studded with tubercles.

A recent case referred by Dr. E. D. Highsmith was one of hematuria, without any symptoms of cystitis. Cystoscopy showed normal bladder mucosa and normal ureter openings. We catheterized the ureters and scarlet urine drained from patient's right kidney, while clear and normal urine came from its fellow and which determined absolutely the source of the hemorrhage.

A more recent case, referred by Dr. C. W. Strickelr, had pain in right side, inner side of right thigh, an intense cystitis, and marked pyuria. Cystoscopy showed a "golf hole" ureter on right and numerous erosions of bladder mucosa.

Ureteral catheterization disclosed a suppurating pyelitis on right, while the urine coming through the other catheter was nor-

CAT-TAILED URETERAL CATHETER IN POSITION IN WELL DISTENDED BLADDER

mal. Urea estimation 2 per cent. Patient consented to a nephrotomy, which I did three weeks ago. The intense cystitis and pain in thigh and kidney region improved but later a nephrectomy became necessary.

A patient referred by Dr. J. S. Todd and Dr. H. F. Harris had recently lost fast in weight. A perineal drainage done by another physician had given him no relief.

I cystoscoped him and found his bladder walls literally hung with long lacy processes and it was with difficulty that I catheterized the ureters, so marked were the changes.

I sent the separate urines from the two kidneys to Dr. Harris, informing him that clinically we had to do with a cancer of the bladder. The doctor found the urine normal.

All three of us agreed that suprapubic cystotomy for permanent drainage was loudly indicated, to which patient consented. I opened the bladder by the high route, gave a specimen of the tumor to Dr. Harris, and established permanent drainage. The doctor pronounced the specimen carcinomatous.

A very interesting case that came into my hands through Dr. Swann, was that of a man 42 years of age. Pain in left kidney region, also near end of penis and in inner side of left thigh with a history of polyuria, and occasionally cloudy urine. Cystoscope showed left *ureter* gaping and injected, bladden mucosa otherwise normal.

A No. 6 *ureteral* catheter was passed easily up to right kidney pelvis, but a like catheter met with obstruction three inches from mouth of left ureter. I then attempted to pass a smaller one, No. 4, but failed.

After trying with a small whalebone or filiform, No. 2, I finally passed the obstruction and as I did so a jet of cloudy urine appeared continuously for about a minute. I decided to leave the filiform in place as long as the patient proved uncomplaining, but after about four hours he complained of severe pain in left testicle and the testis on that side was found drawn clear up to the internal abdominal ring, so the filiform was removed.

This obstruction proved to be a stricture of left ureter. With patience and persistence I have dilated gradually this ureter, once in ten days during a period of five months, up to a No. 8 French. His urine is clear and today the patient states he isfreefrom any pain.

A case sent me last December by Dr. William Perrin Nicolson was that of a man aged 24, who stated he had passed sand and on one occasion had passed a slight amount of blood in his urine. Chief complaint was that of dull pain in left lumbar region. Cystoscopy showed bladder membrane and ureteral openings normal. I passed the ureteral catheters into both ureters and obtained the separate urines from the two kidneys, which I sent to Dr. Paullin, who reported as follows:

"Left kidney secretion profuse with pus cells and right kidney secretion an occasional pus cell. Both specimens contains albumen; no tubercle bacilli."

Nephrotomy on left kidney improved patient for several months, but in June he returned, his chief complain being the cloudy condition of his urine. I catheterized his ureters again and Dr. Paulin reported on the separate specimens:

| Right Kidney. | Left Kidney. |
|---|---|
| Contains few blood cells. | Contains blood cells |
| Roundepithelial cells | Round epithelial cells |
| Pus cells | Pus cells |
| Urea. .007 to cc | Urea .009 to cc |

Left kidney secreting one-tenth amount of urine as its fellow and cellular constituents about same in both specimens. Red cells attributed to slight trauma of ureteral catheters.

By reason of the above findings another operation was contraindicated and the patient was so advised.

Ureteral catheterism, in this instance certainly prevented us from attacking the other kidney and which would have been unwise, and it further showed that the infection was an hematogenous one and had not ascended from the bladder.

928-029 Candler Building.

## Figures and Tables

**Figure f1:**
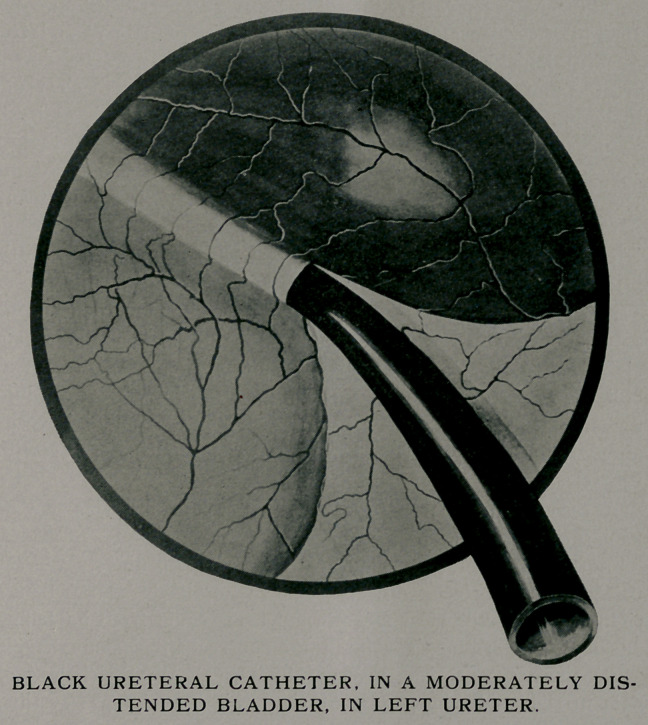


**Figure f2:**